# Pulmonary cysts as a diagnostic indicator of Birt-Hogg-Dubé syndrome in patients with renal neoplasm

**DOI:** 10.1186/s13244-025-02053-y

**Published:** 2025-08-06

**Authors:** Amreen Shakur, Grant D. Stewart, Timothy J. Sadler, Judith L. Babar, Anne Y. Warren, Stephen Scullion, Abhishekh H. Ashok, Sumit Karia, Igor Chipurovski, James Whitworth, Stefan J. Marciniak, Eamonn R. Maher, Maria TA Wetscherek

**Affiliations:** 1 Department of Radiology, Addenbrooke’s Hospital, Cambridge University Hospitals NHS Foundation Trust, Cambridge, UK; 2 Department of Surgery, University of Cambridge, Cambridge Biomedical Campus, Cambridge, UK; 3 Department of Radiology, University of Cambridge and Cambridge University Hospitals NHS Foundation Trust, Cambridge, UK; 4 Department of Pathology, University of Cambridge and Cambridge University Hospitals NHS Foundation Trust, Cambridge, UK; 5 Department of Radiology, West Suffolk Hospital NHS Foundation Trust, Bury St Edmunds, UK; 6 Department of Urology, University of Cambridge, Cambridge Biomedical Campus, Cambridge, UK; 7 Department of Medical Genetics, University of Cambridge and NIHR Cambridge Biomedical Research Centre, Cambridge, UK; 8 Respiratory Medicine, Addenbrooke’s Hospital, Cambridge University Hospitals NHS Foundation Trust, Cambridge, UK; 9 Cambridge Institute for Medical Research (CIMR), University of Cambridge, Cambridge, UK; 10 Respiratory Medicine, Royal Papworth Hospital NHS Foundation Trust, Cambridge, UK; 11 Aston Medical School, Aston University, Birmingham, UK

**Keywords:** Birt-Hogg-Dubé syndrome, Computed tomography, Renal cancer, Cystic lung disease

## Abstract

**Objectives:**

To assess the presence and CT features of pulmonary cysts (PCs) in patients with renal neoplasms (RN) as a hallmark of Birt-Hogg-Dubé syndrome (BHDS).

**Materials and methods:**

Single institution retrospective study of all patients with histological RN between May 2014 and May 2020. Individuals with non-renal neoplasm, nephroblastoma, benign cysts, < 18 years old, or without thoracic CT were excluded. Demographics, history of smoking, pneumothorax and cutaneous fibrofolliculomas/trichodischomas, family history of pneumothorax or RN, and genetic testing were recorded. Number, location, distribution and morphology of PCs were assessed on thoracic CT. Differences between patients with positive (BHD+) and negative (BHD−) genetics were analysed. An independent cohort of 10 BHDS patients was added to calculate the diagnostic accuracy of cyst features.

**Results:**

Of 1475 patients with RN, 127 (8.6%) had PCs; 40 underwent genetic testing (median age 56 [49–68], 28 men), and 6/127 (4.7%) individuals tested positive for BHDS. BHD+ had significantly more and larger cysts, affecting more lobes (*p* < 0.01). Higher prevalence of PCs with a perivascular (100% vs. 37%; *p* = 0.01) and interlobular septal location (100% vs. 16%; *p* < 0.001), and perilymphatic distribution (100% vs. 5%; *p* < 0.001) was found in BHD+. All BHD+ had elliptical, irregular, and variable shape PCs, compared to a lower prevalence of these in BHD− (*p* < 0.01). Traversing vein sign was more common in BHD+ (83% vs. 24%; *p* = 0.01). The highest accuracy was achieved for perilymphatic distribution (97%), followed by irregular shape (94%) and interlobular septal location (91%).

**Conclusion:**

Specific CT features of PC in patients with RN can be highly indicative of BHDS.

**Critical relevance statement:**

Radiologists can play a crucial role in the diagnosis of Birt-Hogg-Dubé syndrome (BHDS) by recognising specific CT features of pulmonary cysts; a diagnosis of BHDS has implications for family testing and timely, life-long screening for renal neoplasm.

**Key Points:**

- Birt-Hogg-Dubé syndrome (BHDS) should be considered in patients with renal neoplasms and multiple pulmonary cysts.
- A lower zone predominant, perilymphatic distribution of pulmonary cysts is a strong indicator of BHDS.
- Identifying specific CT features of pulmonary cysts can improve recognition of BHDS.

**Graphical Abstract:**

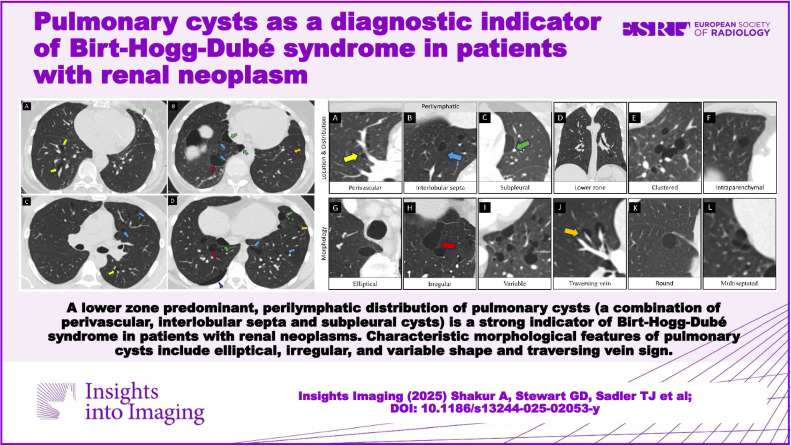

## Introduction

Birt-Hogg-Dubé syndrome (BHDS) is a rare autosomal dominant condition, caused by germline mutations in the *FLCN*-gene, which encodes the tumour suppressor protein folliculin [1–3]. Its major manifestations are cutaneous fibrofolliculomas/trichodischomas (FF/TD), pulmonary cysts (PCs) with increased risk of spontaneous pneumothorax (SPTX) and renal neoplasms (RNs) [1].

The exact prevalence of BHDS is unknown, as the condition is generally considered underdiagnosed because of its variability in clinical expression, with only one or any combination of the pulmonary, cutaneous or renal manifestations of the disease potentially being present [1, 4–6]. The 2024 clinical practice guideline for BHDS makes a strong recommendation to consider a potential diagnosis of BHDS if any combination of multiple FF/TD, PCs or RN presents in the same individual [1]. A clinical diagnosis of BHDS can be made if two minor criteria from the European BHD Consortium are met [3], including: bilateral basally located pulmonary cysts with no other apparent cause; early onset (< 50 years), multifocal or bilateral RN, or histological hybrid oncocytic renal tumour (HOT) [1, 3]. However, the absence of these renal criteria does not exclude a diagnosis of BHDS, as patients can present with a single RN, of any histological subtype and at ages > 50 years [1, 7–9]. Lack of awareness by clinicians and radiologists can delay life-saving screening for renal cancer and family testing by several years [1, 8].

PCs are the most penetrant aspect of the phenotype across populations, seen in 80–100% of individuals with BHDS, and are associated with SPTX in 24–38% of patients [10–12]. Characteristically, the cysts are mostly located below the carina [10]. These are frequently misdiagnosed as spontaneous blebs, bullae or emphysema [13]. Several CT characteristics of the PCs have been reported in the literature; however, only multiple, bilateral and lower zone predominance have been included in the guidelines to date [1, 3]. Histopathological studies demonstrate a subpleural, perivascular and interlobular septal location of PCs in BHDS [13]. A similar distribution is defined at CT as a perilymphatic distribution [14]. We hypothesised that a perilymphatic distribution of PCs on thoracic CT can indicate a diagnosis of BHDS.

The purpose of our study was to assess the presence of PCs in patients with RN and to evaluate which characteristics of the PCs are highly indicative of BHDS, with particular focus on cyst distribution.

## Materials and methods

### Study design and population

**Fig. 1:**
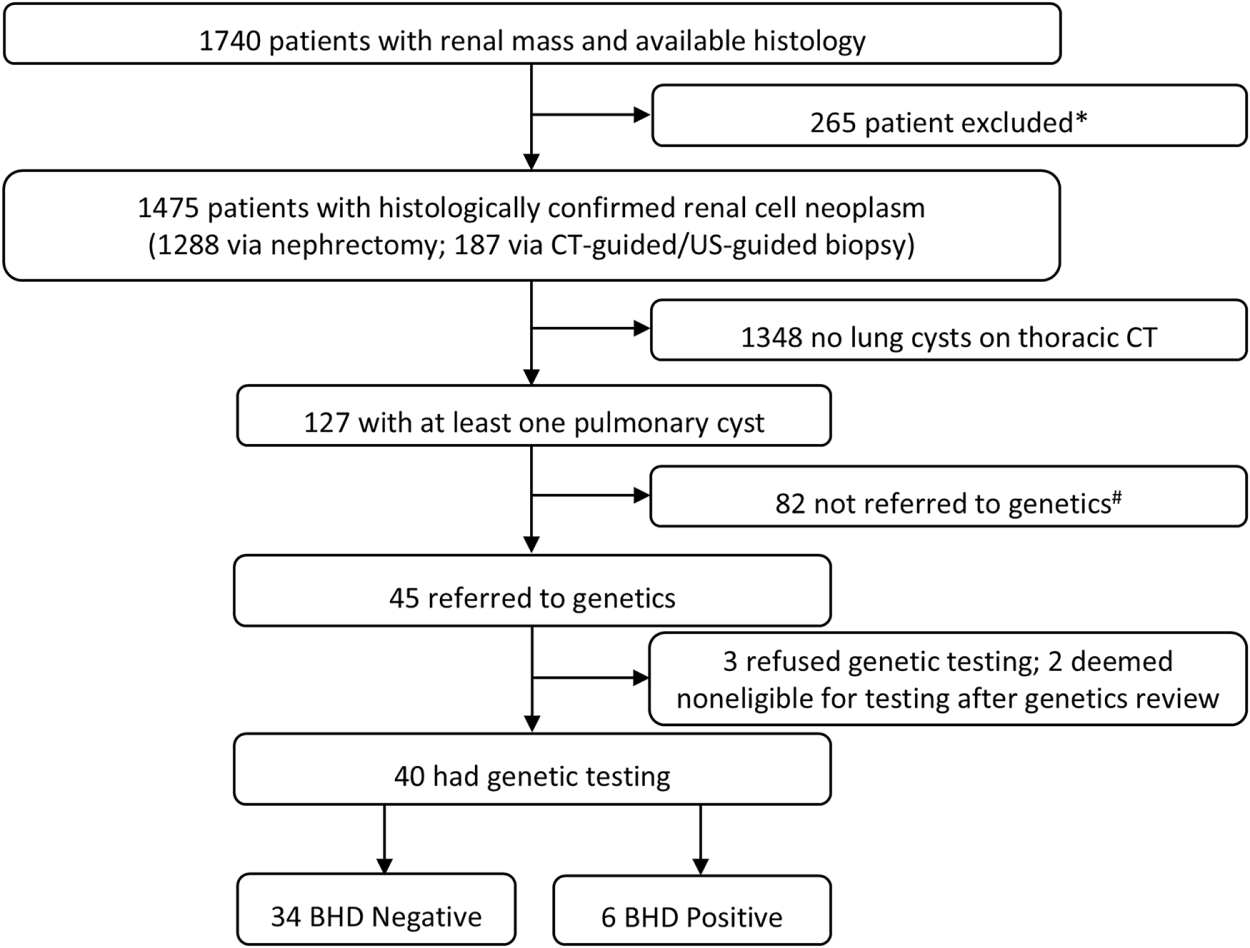
Flowchart of the study cohort. * 265 patients initially excluded: 27 under 18 years, 189 non-renal cell tumours (146 urothelial carcinoma, 14 lymphoma, 14 angiomyolipoma, 15 metastasis), 4 nephroblastoma, 16 benign cyst, 29 had no thoracic CT; ^#^ 82 not referred to genetics due to being too unwell, deceased soon after diagnosis (*N* = 34), or did not answer invitation for genetics clinic review and living out of area

This was a single-centre retrospective observational study performed in a tertiary referral centre. Using a computerised search of our hospital electronic records, we included all patients who underwent histological assessment following nephrectomy or image-guided biopsy for suspected RN between May 2014 and May 2020. Patients with non-renal neoplasm, nephroblastoma (has not been described in BHDS), benign cysts, < 18 years old, and no thoracic CT were excluded. Figure 1 displays a detailed flowchart of the study population.

Ethical committee approval and written informed consent were waived because this was a retrospective service evaluation study registered with our institution’s Quality and Safety Information System (ID 2961 with registration number PRN8961), with board approval to publish obtained in January 2025. We included an additional independent cohort of genetically confirmed BHDS patients recruited in the Molecular Pathology of Human Genetic Disease study, with South Birmingham Research Ethics Committee approval (IRAS ID 50895) and informed consent obtained. The study adhered to the principles of the Declaration of Helsinki.

### Imaging methods

The thoracic CT scans were performed at our institution on one of five scanners (Siemens Emotion 16, Siemens Sensation 64, Somatom Definition AS, Somatom Definition AS+, Somatom Definition Flash) and at 7 district general hospitals or private providers on different scanners (GE Medical Systems Optima CT660, Toshiba Aquilion Prime, Siemens Somatom Force), using volumetric acquisition from lung apices to the diaphragm with breath-hold at the end of deep inspiration. The scan parameters for the protocol used at our institution included 0.6 mm collimation, 1.3 pitch, 0.5 s/rotation, 300–409 mm field of view and 512 × 512 matrix, 120 [120–130] kV and mA adjusted according to patient habitus. All examinations were reconstructed iteratively as contiguous 1- or 2-mm thick slices.

### Data analysis

The initial CT thorax of each patient was assessed for the presence of PCs by three board-certified radiologists, with 1, 3, and 4 years of consultant-level experience, respectively. A PC was defined as per Fleischner Society glossary of terms for thoracic imaging, as “a circumscribed and well-defined radiolucency and/or low attenuation area with a thin wall” [15]. Patients without PCs were excluded from subsequent analysis. For patients with at least one PC, we documented demographics (age, gender, ethnicity), smoking status (never-smoker or ever-smoker, the latter including current or former smoker), as well as personal and family history of pneumothorax or RN and presence of skin lesions (FF/TD). Genetic testing results and histological tumour subtype were recorded. We categorised each patient as having at least two BHDS criteria available at the time of CT review if at least 2 of the following were present: age < 50 years at diagnosis of RN, multiple RN, multiple PCs. RN location and the presence of multifocal or bilateral RN were recorded from baseline abdominal CT reports.

**Fig. 2:**
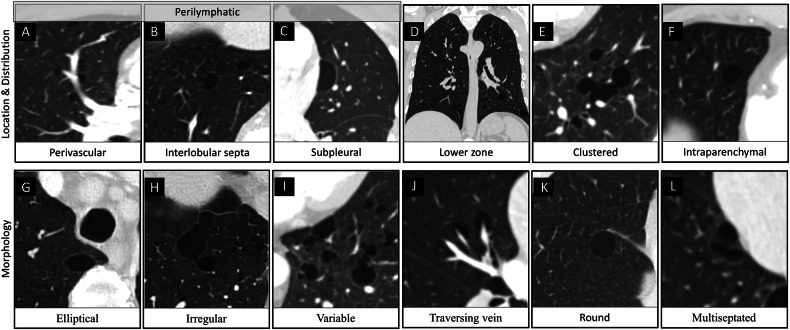
CT characteristics of pulmonary cysts: *perivascular location* (axial plane, **A**); *interlobular septal* location (axial plane, **B**); *paramediastinal location* (axial plane, **C**); *perilymphatic-predominant* distribution (**A**–**C**); *lower zone predominant* distribution (coronal reformat, **D**); *clustered* (axial plane, **E**); *intraparenchymal* location (axial plane, **F**); *elliptical-shape* morphology (axial plane, **G**); *irregular-shaped* morphology (axial plane, **H**); *variable* morphology (axial plane, **I**); *traversing vein* sign (axial plane, **J**); *round-shaped* morphology—a symmetric cyst with a diameter ratio of long to short axes < 1.1 (axial plane, **K**); *multiseptated cyst* (axial plane, **L**). Note that all cysts are thin-walled

The CT characteristics of PCs were assessed on the lung window reconstruction by a thoracic radiologist with 6 years of experience, blinded to patients’ clinical data. Cyst features analysed were: total number of cysts, largest cyst greatest diameter and location, number of affected lobes, individual cyst location (*subpleural*—a cyst abutting the pleura, including *paramediastina*l pleura; *parenchymal*—a cyst entirely surrounded by lung parenchyma; *perivascular*—a cyst abutting the pulmonary vessels; *interlobular* *septal* *location*—a cyst located along the interlobular septa with the longest axis continuing with an interlobular septum); cysts overall distribution (*lower zone predominance*—majority of the cysts below the carina; *perilymphatic distribution*—a combination of cysts with subpleural, perivascular and interlobular septal location; *laterality-predominance*; presence of *clustered cysts*—cysts which appear grouped together in one area); and cysts morphology (*round shape*—a symmetric cyst with a diameter ratio of long to short axes < 1.1; *elliptical shape*—a symmetric cyst with a diameter ratio of long to short axes > 1.1; *irregular shape*—a cyst with a shape other than round or elliptical; *variable morphology*—presence of different shapes of cysts: round, elliptical, irregular; *thin wall; multiseptated*—cyst with multiple septations; *traversing vein sign*—a vein crossing through the inside of the cyst). Cyst features are illustrated in Fig. 2. Analysis of location and distribution was performed only on cases with at least 5 PCs, while all other analyses were performed on all cases. This was because < 5 PCs may be seen with ageing [16].

### Statistical analysis

Data analysis was performed using R statistical software version 4.0.5 (R Foundation for Statistical Computing, Vienna, Austria). The demographic, clinical and PCs characteristics are presented as absolute numbers and percentages for categorical variables, and median and interquartile range (IQR) for continuous variables. Differences between patients with positive (BHD+) and negative (BHD−) genetics were analysed. Fisher’s exact test and Wilcoxon rank-sum test were used for comparing categorical or continuous variables, respectively, between the BHDS positive groups (BHD+, independent cohort) and the BHD− group. Sensitivity, specificity, positive and negative predictive value, and accuracy were calculated for location, distribution and morphology features based on all BHDS cases and BHD− cohort. All tests were 2-sided, with a significance level of 0.05.

## Results

Of the 1740 patients with renal mass and available histology, 1475 were included for CT analysis. Of these, 127 (8.6%) had PCs and 56 (44%) had at least 5 PCs. Forty patients underwent genetic testing (31.5%; median age 56 [49–68], 28 men), with 6 (4.7% of those having any cysts) testing positive for BHDS (15% of those tested). Detailed demographic and clinical features are presented in Table 1. FF/TD and patient history of SPTX were only present in BHD+. Family history of pneumothorax was more common in BHD+ than in BHD− (3 (50%) vs. 1 (4%); *p* = 0.02), while family history of RN and presence of two diagnostic criteria did not differ significantly. None of the non-tested patients had two criteria (they were either older than 50 years or had a single non-HOT).

**Table 1:** Demographic and clinical features of the study and validation cohorts

Parameter	Total *N* = 40 (%)	BHD+^a^ *N* = 6 (15%)	BHD−^b^ *N* = 34 (85%)	Independent BHDS cohort (*N* = 10)	*p*-value* {a-b}
Age (years)	56 [49–68]	67 [62–68]	53 [47–66]	47 [38–57]	0.18
Gender (male)	28 (70%)	4 (67%)	24 (71%)	2 (20%)	0.85
Ethnicity (white British)	32 (84%)	6 (100%)	26 (81%)	8 (80%)	0.56
Smoking history (never-smoker)	15 (43%)	5 (83%)	10 (33%)	10 (100%)	**0.009**
At least 2 criteria^#^ (yes)	16 (40%)	4 (67%)	12 (35%)	1 (10%)	0.20
SPTX (yes)	4 (13%)	4 (67%)	0 (0%)	4 (40%)	**< 0.001**
Skin lesions^$^ (yes)	6 (19%)	6 (100%)	0 (0%)	5 (50%)	**< 0.001**
FHx PTX (yes)	4 (13%)	3 (50%)	1 (4%)	6 (60%)	**0.02**
FHx RN (yes)	3 (10%)	1 (17%)	2 (8%)	4 (40%)	0.49

Missing data excluded from the percentages: 2 ethnicity (all BHD−); 5 smoking history (1 in BHD+); 8 history of PTX (all BHD−); 9 FF/TD (all BHD−); 10 family history of PTX and RN, respectively (9 in BHD− and 1 in the independent cohort)

*SPTX* spontaneous pneumothorax, *FHx* family history, *RN* renal neoplasm

* From comparing groups with RN with and without BHD (statistically significant differences marked in bold)

^#^ At least 2 criteria identifiable on CT—age < 50 years at diagnosis of RN, multiple RN, multiple lung cysts

^$^ Skin lesions—fibrofolliculoma/trichodischoma (FF/TD)

^a^ BHD+: cohort with renal neoplasm and Birt-Hogg-Dubé syndrome confirmed by genetic testing

^b^ BHD−: cohort with renal neoplasm and negative genetic testing result for BHD

**Fig. 3:**
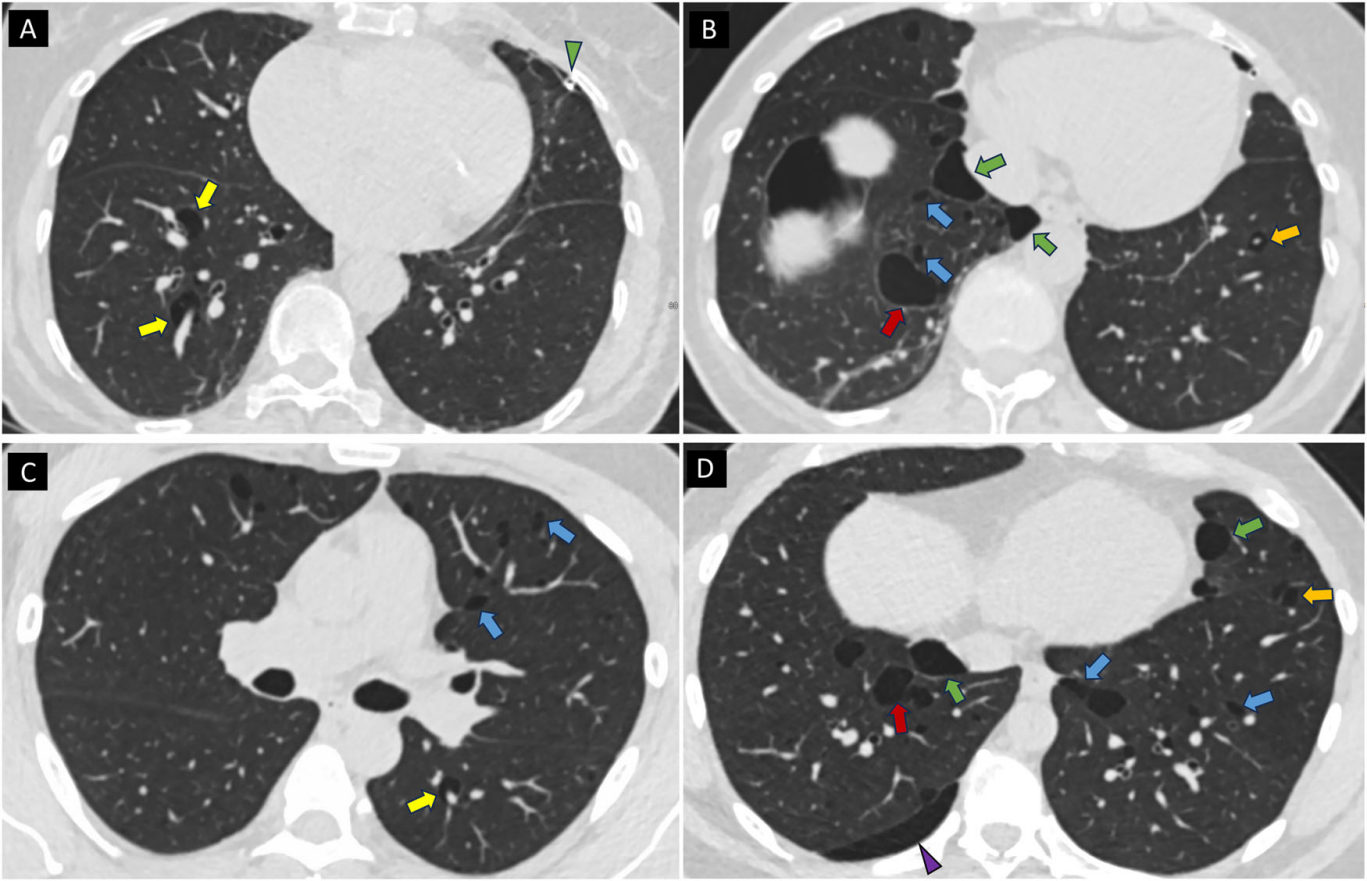
Two genetically confirmed Birt-Hogg-Dubé syndrome cases. Axial images (**A**, **B**) of a 70-year-old female presenting with a personal and family history of recurrent spontaneous pneumothorax (SPTX) and facial fibrofolliculomas. She was diagnosed with clear cell renal cancer following screening for renal neoplasia and subsequent partial nephrectomy. Axial images (**C**, **D**) of a 39-year-old male, included in the BHDS independent cohort, who presented with recurrent SPTX and facial fibrofolliculomas. Both cases demonstrate lower zone predominant cysts with a perilymphatic distribution, specifically perivascular cysts (yellow arrows), subpleural paramediastinal cysts (green arrows), and interlobular septal cysts (blue arrows). Cysts with variable size and shape and several with irregular shape (red arrows) are present. Cysts with traversing vein are demonstrated in the left lower lobe (orange arrows). Note is made of a left-sided chest drain (green arrowhead) and a small right pneumothorax (purple arrowhead)

Detailed characteristics, including specific mutation of each BHD+ patient, are presented in Table 2. Three patients (50%) had a single renal tumour. The histological subtype of RN in patients with BHDS in our study was: 2 (33%) HOT, 1 (17%) oncocytoma, 1 (17%) chromophobe renal cell carcinoma (RCC), 1 (17%) papillary RCC and 1 (17%) clear cell RCC. Two patients (cases 3 and 4, one presented in Fig. 3) had metastatic renal cancer and died within 2 years of diagnosis. Four patients (66.7%) had a history of SPTX; the two patients without SPTX had the largest cysts, measuring 22 mm and 24 mm, respectively.

**Table 2:** Characteristics of the 6 cases with genetically confirmed Birt-Hogg-Dubé syndrome

Case	Age	Sex	RN number	RN histology	PTX	FF/TD	FHx	*FLCN-*mutation
1	50	M	single	Oncocytoma	No	Yes	PTX, RN	c.1245C>A p.Cys415*
2	68	M	multiple bilateral	ChRCC	Yes	Yes	No	c.1657T>C p.(Trp553Arg)
3	61	M	single	pRCC type 2	Yes	Yes	PTX	c.1285dup p.(His429Profs*27)
4	70	F	single	ccRCC	Yes	Yes	PTX	c.1285dupC, p.(His429Profs*27)
5	68	F	bilateral	HOT	Yes	Yes	No	c.1177-5_1177-3delCTC
6	66	M	multiple bilateral	HOT	No	Yes	No	c.57_58delCT p.(Phe20Leufs*16)

*RN* renal neoplasm, *PTX* pneumothorax, *FF/TD* fibrofolliculoma/trichodischoma, *FHx* family history, *ChRCC* chromophobe renal cell carcinoma, *pRCC* papillary RCC, *ccRCC* clear cell RCC, *HOT* hybrid oncocytic tumour

All cases had lung cysts. Age: age at diagnosis of RN

Less than 5 cysts were found in 15 (37.5%) of genetically tested cases—all BHD−, and 57 (66%) cases without genetic testing. Detailed analysis of PCs in the BHD+, BHD− and independent BHDS cohort is presented in Table 3; a similar analysis on the cohort without genetic testing is presented in Supplementary Table 1. Patients with BHD had significantly more cysts (median 63 vs. 5; *p* = 0.002), more affected lobes (median 6 vs. 2; *p* = 0.002), and larger cysts (median diameter 25 mm vs. 11 mm; *p* = 0.0007); the differences remained significant after excluding cases with < 5 cysts (*p* = 0.02; *p* = 0.001).

**Table 3:** Characteristics of lung cysts on CT in patients with renal neoplasm

Parameter	BHD+^a^ (N* = 6)	BHD−^b^ (*N* = 34)	Independent BHDS cohort (*N* = 10)	*p*-value {a-b}
**Number of cysts** median [IQR]	63 [52–84]	5 [1–18]	57 [35–75]	**0.002**
After exclusion^$^	63 [52–84]	16 [6–41]	57 [35–75]	**0.02**
< 5	0	15	0	
5–20	1	11	1	
> 20–40	5	8	9	
**Number of affected lobes**	6 [6]	2 [1–5]	6 [6]	**0.002**
After exclusion^$^	6 [6]	5 [4–6]	6 [6]	**0.02**
**Cyst location** *N* (%)				
Subpleural	6 (100%)	13 (68%)	10 (100%)	0.28
Paramediastinal	6 (100%)	9 (43%)	10 (100%)	**0.50**
Perivascular	6 (100%)	7 (37%)	10 (100%)	**0.01**
Interlobular septa	6 (100%)	3 (16%)	10 (100%)	**< 0.001**
Intraparenchymal	6 (100%)	14 (74%)	10 (100%)	0.29
**Overall distribution** *N* (%)				
Perilymphatic predominant^α^	6 (100%)	1 (5%)	10 (100%)	**< 0.001**
Lower zone predominant	6 (100%)	14 (74%)	10 (100%)	0.29
More in one lung	1 (17%)	4 (21%)	1 (10%)	0.81
Clustered	3 (50%)	0 (0%)	2 (20%)	**0.009**
**Largest cysts**				
Largest diameter (mm)	25 [23–29]	11 [8–15]	23 [18–29]	**< 0.001**
After exclusion^$^	25 [23–29]	11 [10–15]	23 [18–29]	**0.001**
Lobe N (After exclusion^$^)				
RUL	0 (0)	6 (4)	1 (1)	0.59 (0.40)
RML	1 (1)	3 (2)	1 (1)	
RLL	2 (2)	5 (1)	3 (3)	
LUL	1 (1)	6 (3)	0 (0)	
Lingula	1 (1)	2 (2)	1 (1)	
LLL	1 (1)	12 (7)	4 (4)	
**Cyst morphology** *N* (%)				
Shape after exclusion^$^				
Round	6 (100%)	19 (100%)	10 (100%)	-
Elliptical	6 (100%)	4 (21%)	10 (100%)	**0.001**
Irregular	6 (100%)	2 (11%)	10 (100%)	**< 0.001**
Variable	6 (100%)	5 (26%)	10 (100%)	**0.003**
Thin wall	6 (100%)	34 (100%)	10 (100%)	-
Traversing vein	5 (83%)	8 (24%)	5 (50%)	**0.01**
Multiseptated	6 (100%)	33 (97%)	100 (100%)	-

* *N*—number of patients

^$^ Total cyst number < 5 excluded from analysis of location and distribution (*N* = 15)

^α^ Perilymphatic predominant—combination of subpleural, along the interlobular septa and perivascular cysts

^a^ BHD+: cohort with renal neoplasm and Birt-Hogg-Dubé syndrome confirmed by genetic testing

^b^ BHD−: cohort with renal neoplasm and negative genetic testing result for BHD

Statistically significant differences marked in bold

Cyst location differed significantly, with a higher prevalence of perivascular (100% in BHD+ vs. 37% in BHD−; *p* = 0.01) and interlobular septal (100% vs. 16%; *p* = 0.0005) locations. All BHD+ patients exhibited perilymphatic distribution (100% vs. 5%; *p* < 0.0001). Morphological analysis showed that all BHD+ patients had cysts exhibiting elliptical, irregular, and variable shapes, compared to a lower prevalence of these features in BHD− (*p* = 0.001–0.003). Traversing vein sign was more common in BHD+ (83% vs. 24%; *p* = 0.01), but multiseptated cysts were similarly encountered in both groups (100% vs. 97%). Two illustrative BHDS cases are presented in Fig. 3.

**Fig. 4:**
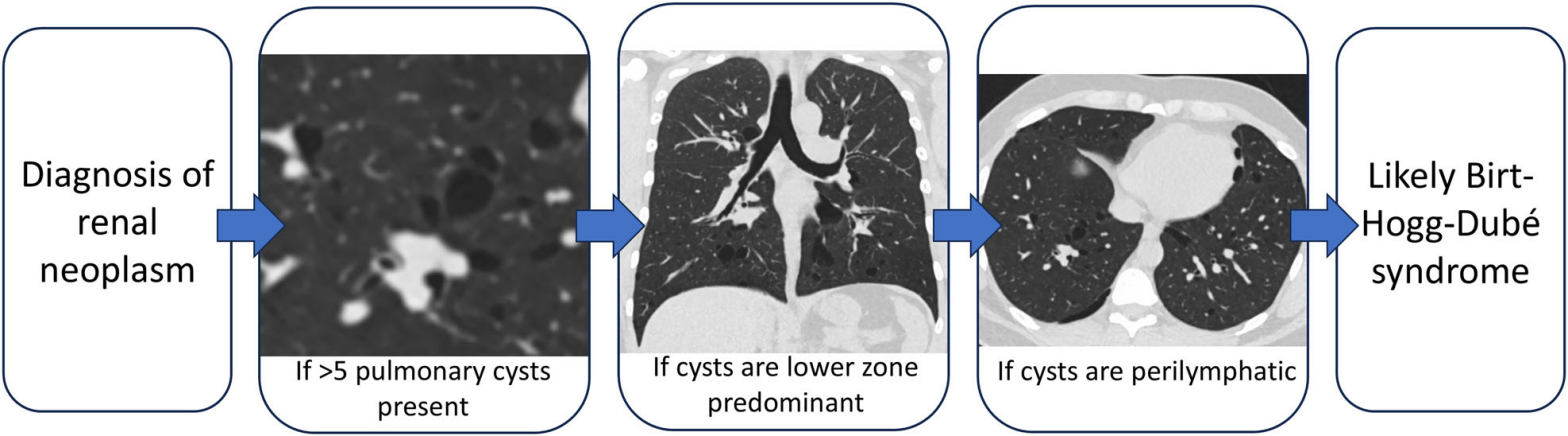
Proposed systematic approach to consider a diagnosis of Birt-Hogg-Dubé syndrome based on CT features of pulmonary cysts following diagnosis of renal neoplasm. We define perilymphatic cysts as a combination of perivascular, subpleural and interlobular septal cysts

Among CT features of location, distribution and morphology, the highest accuracy was achieved by perilymphatic distribution (97%), followed by the presence of irregularlyshaped cysts (94%) and cysts with an interlobular septal location (91%), as shown in Table 4. We propose a radiological systematic approach to consider genetics referral based on CT features of PCs following diagnosis of RN in Fig. 4.

**Table 4:** Diagnostic value of cysts location, distribution and morphology in Birt-Hogg-Dubé syndrome

Parameter	BHD (N* = 16)	BHD− (*N* = 34)	Se/Sp/PPV/NPV	Accuracy (95% CI)	*p*-value
**Cyst location** *N* (%)					
Subpleural	16 (100%)	13 (68%)	100/32/55/100	63 (46–77)	**0.02**
Paramediastinal	16 (100%)	9 (43%)	100/53/64/100	74 (58–86)	**< 0.001**
Perivascular	16 (100%)	7 (37%)	100/63/70/100	80 (64–90)	**< 0.001**
Interlobular septa	16 (100%)	3 (16%)	100/84/84/100	**91** (**78–97)**	**< 0.001**
Intraparenchymal	16 (100%)	14 (74%)	100/26/53/100	60 (44–74)	0.05
**Overall distribution** *N* (%)					
Perilymphatic predominant^α^	16 (100%)	1 (5%)	100/95/94/100	**97** (**85–99)**	**< 0.001**
Lower zone predominant	16 (100%)	14 (74)	100/26/53/100	60 (44–74)	0.05
More in one lung	2 (13%)	4 (21%)	13/79/33/52	49 (33–64)	0.67
Clustered	5 (31%)	0 (0%)	31/100/100/63	69 (52–81)	**0.01**
**Cyst morphology** *N* (%)					
Shape after exclusion^$^					
Round	16 (100%)	19 (100%)	100/0/46/-	46 (31–62)	-
Elliptical	16 (100%)	4 (21%)	100/79/80/100	89 (74–96)	**< 0.001**
Irregular	16 (100%)	2 (11)	100/89/89/100	**94** (**81–98)**	**< 0.001**
Variable	16 (100%)	5 (26%)	100/74/76/100	86 (71–94)	**< 0.001**
Traversing vein	10 (63%)	8 (24%)	63/76/56/81	72 (58–83)	**0.01**
Multiseptated	16 (100%)	33 (97%)	100/3/33/100	34 (22–48)	-

*BHD* Birt-Hogg-Dubé syndrome confirmed by genetic testing, *BHD−* negative genetic testing result for BHD, *Se/Sp/PPV/NPV/ACC* sensitivity/specificity/positive predictive value/negative predictive value/accuracy for a genetically confirmed diagnosis of BHDS in (highest values highlighted in bold)

* *N*—number of patients

^$^ Total cyst number < 5 excluded from analysis of location and distribution *(N* = 15)

^α^ Perilymphatic predominant—subpleural, along the interlobular septa and perivascular cysts

## Discussion

BHDS is a rare condition that is generally underdiagnosed. Here we present the largest RN cohort evaluated for PCs as a marker of BHDS, with special focus on cyst morphology and distribution. We found that: (1) 8.6% of patients with RN had PCs and, among those with RN and PCs, 4.7% had genetically confirmed BHDS; (2) all patients with genetically confirmed BHDS had lower zone, perilymphatic-predominant PCs; (3) perilymphatic distribution (a combination of perivascular, interlobular septal and subpleural cysts) was the most specific feature of BHDS; (4) characteristic morphological features of PCs in BHDS included: elliptical, irregular and variable shape, and traversing vein sign. While previous studies reported cysts with subpleural and perivascular location in BHDS, this is the first study to highlight the perilymphatic distribution as specific for BHDS. This could be due to our assessment of all 3 individual cyst locations, including interlobular septal cysts, which was the third most specific feature of BHDS in this study.

Pulmonary involvement is the most common manifestation in BHDS, with the majority of the cyst burden below the carina [10]. Thoracic high-resolution CT (HRCT) is the modality of choice for the diagnosis of cystic lung disease and allows for the differential diagnosis, which includes BHDS, lymphangioleiomyomatosis (LAM), pulmonary Langerhans cell histiocytosis, lymphocytic interstitial pneumonia (LIP) and others [17–19]. Thoracic HRCT can be offered to asymptomatic patients diagnosed with BHDS after the age of 20 years to assess for the presence of PCs [1].

The distribution of PCs has been shown to be a useful discriminator among cystic lung diseases. Whilst a diffuse pattern of cysts is more frequently observed in LAM, a lower zone predominance of PCs is more commonly seen in BHDS and LIP [19]. Cysts in LIP have been described with a lower zone predominance and a random distribution [18, 19]. Similar to perilymphatic nodules [14], a perilymphatic distribution of the PCs typically encompasses subpleural, interlobular septal, and a bronchovascular bundle location. The pathogenesis of PCs in BHDS is not yet fully understood, but their relationship to the visceral pleura, interlobular septa and bronchovascular bundles has been described in histopathological studies; these have indicated that BHD-related cysts are initially located very close to the interlobular septa and/or are subpleural [13], which supports our observation. BHD cysts have been described typically in the hilar or central region of the lung [12] as well as the subpleural and paramediastinal regions [10]. This different approach to cyst distribution showed inconsistent results in the literature, with studies showing more peripherally distributed cysts [20], cysts predominantly in the medial zones [21], or neither central nor peripheral predominance [22]. Some perivascular cysts may encase a pulmonary vein, resulting in the traversing vein sign, also known as “air-cuff” sign [20]. This was a significant finding in our BHD+ cohort. Cysts with protruding veins have been described as a hallmark feature of BHDS [13, 23], aligning with our findings.

We also observed a high prevalence of elliptical cyst morphology, which has been previously described as a useful discriminator between cystic lung diseases and as a characteristic feature in BHDS patients [10]. Furthermore, all patients with BHDS demonstrated cysts with variable and irregular morphologies, with a significant difference observed between groups with and without BHDS in our study, further corroborating findings from previous studies [21, 24, 25]. Other cyst patterns, including an intraparenchymal [4] and multiseptated predominance [22] have been described in the literature as commonly found in BHDS patients; however, we found these to be overall highly prevalent with no significant difference to BHD− cases.

BHDS patients displayed a significantly higher number of PCs, and the maximum size of PCs in these patients was notably larger compared to the BHD− group, consistent with findings reported in the literature [21, 26]. Previous reports showed that solitary cysts may be found in BHDS, and the absence of multiple cysts does not exclude BHDS [27, 28]. In our study, no patient with < 5 cysts had BHDS, and it is worth noting that a few incidental lung cysts may be normal for age [16].

The most common presentation of pulmonary involvement with BHDS is the occurrence of SPTX. Recently, a 28.4–37.3% lifetime risk of pneumothorax in *FLCN*-mutation carriers was reported to age 65 years [29]. Both personal and family history of pneumothorax were significant amongst our BHD+ cohort. Previous studies reported CT features of PCs in BHDS associated with history and number of recurrent SPTXs, including total lung cyst volume and largest cyst diameter and volume [26]; specifically, a maximum diameter < 29 mm had a significantly lower risk of SPTX [30]. Indeed, the two BHD+ patients in our study who had not experienced a pneumothorax had their largest cyst below this size. A recent study validating the results of a previous meta-analysis on recurrence of SPTX showed that the size of contralateral cysts is associated with a higher risk of contralateral recurrence in young individuals [31]. Furthermore, PCs are thought to remain stable in size based on the results of three small cohort studies that serially imaged BHDS patients for a period ranging between 1 and 66 months [10]. Progression analysis was not an objective of our study; however, patients with BHDS and RN offer a unique opportunity to perform such an evaluation since they receive regular surveillance imaging that often includes thoracic CT. While renal imaging for RN screening is recommended every 1–2 years, preferably annually and using MRI, monitoring of PCs with repeated HRCT in BHDS is generally unnecessary [1].

Our study has several limitations. First, despite reviewing a relatively large number of RN cases, the number of genetically confirmed BHDS patients is relatively low. BHDS is a rare disease, with pathogenic *FLCN*-variants estimated to affect only 1 in 4190 individuals in the UK Biobank [29]. To mitigate this limitation, an additional independent cohort of BHDS patients was included for cyst analysis. While the study was conducted at a single institution, this was a tertiary referral centre with cases referred from seven other hospitals. A further limitation is the potential for false-negative genetic results. While we considered genetic results as a reference standard, the reported detection rate of *FLCN-*mutation in individuals diagnosed with BHDS is estimated to be 88–96% [1]. A false negative genetic test is therefore unlikely, particularly in the absence of other clinical features such as SPTX and cutaneous FF/TD. One patient with lower zone and perilymphatic-predominant distribution of PCs had negative genetics, albeit no other clinical feature of BHDS was documented in this case. We acknowledge that, due to its retrospective nature, the data gathered was limited by the available medical records. Lastly, a significant number of patients with PCs and RN had no genetic testing, with the majority being too unwell or deceased soon after diagnosis of RN, but most of these had < 5 PCs. On review of CT features of the cases with no genetics result, no other patient had perilymphatic-predominant distribution. Further studies are needed to validate our findings in patients with BHDS without RN, as well as other cohorts with BHDS and RN, as there is a growing interest in identifying phenotypes strongly associated with SPTX or RN. Furthermore, studies such as this could inform the Genomic Test Directory [32], which guides eligibility for genetic testing, to optimise criteria for genetic testing in BHDS. Finally, in patients with incidental pulmonary cysts suggestive of BHD, careful review of the renal parenchyma included on thoracic imaging is recommended, as it could lead to early identification of RN.

In conclusion, our study demonstrates that specific CT features of PCs can serve as diagnostic indicators for suspecting BHDS in patients with RN. Notably, we highlight the perilymphatic-predominant distribution as a distinctive feature of BHDS. We underscore the important role that radiologists can play in the diagnosis of BHDS and support the current guidelines of recommending genetics referral with a view for genetic testing of those patients with features of BHDS, to facilitate prompt family testing and timely, life-long screening for RN.

## Supplementary information

ELECTRONIC SUPPLEMENTARY MATERIAL

## Data Availability

The datasets analysed during the current study are available from the corresponding author on reasonable request.

## Abbreviations

BHD−: *FLCN*-mutation negative
BHD+: Birt-Hogg-Dubé *FLCN*-mutation positive
BHDS: Birt-Hogg-Dubé syndrome
FF: Fibrofolliculoma
FLCN: Folliculin
HOT: Hybrid oncocytic tumour
LAM: Lymphangioleiomyomatosis
LIP: Lymphocytic interstitial pneumonia
PCs: Pulmonary cysts
RCC: Renal cell carcinoma
RN: Renal neoplasm
SPTX: Spontaneous pneumothorax
TD: Trichodiscoma
