# Supplementary material for: Pulmonary cysts as a diagnostic indicator of Birt-Hogg-Dubé syndrome in patients with renal neoplasm
Source: Insights Imaging. 2025 Aug 6;16:169. doi: 10.1186/s13244-025-02053-y (PMC12328879; doi:10.1186/s13244-025-02053-y)
Supplement: Supplementary file 1 — ELECTRONIC SUPPLEMENTARY MATERIAL [file 13244_2025_2053_MOESM1_ESM.pdf]

Pulmonary cysts as a diagnostic indicator of Birt-Hogg-Dubé syndrome in patients with renal neoplasm

ELECTRONIC SUPPLEMENTARY MATERIAL

Insights Imaging (2025) Shakur A, Stewart GD, Sadler TJ, et al.

Table 1. Characteristics of lung cysts on CT in patients with renal neoplasm but no genetic testing versus patients diagnosed with Birt-Hogg-Dubé syndrome

Parameter	BHDS[a] (N*=16)	Not tested cohort[b] (N=87)	P-value
**Number of cysts**$^{\$}$ median [IQR]	60 [44-78]	11 [6-18]	**<0.001**
<5	0	57	
5-20	2	24	
>20	14	6	
**Number of affected lobes**$^{\$}$	6 [6-6]	4 [3-5]	**<0.001**
**Largest cyst diameter** (mm)	25 [19-30]	11 [9-19]	**<0.001**
**Cyst location** N (%)			
Subpleural	16 (100%)	18 (58%)	**0.002**
Paramediastinal	16 (100%)	9 (29%)	**<0.001**
Perivascular	16 (100%)	10 (32%)	**<0.001**
Interlobular septa	16 (100%)	4 (13%)	**<0.001**
Intraparenchymal	16 (100%)	100 (100%)	-
**Overall distribution** N (%)			
Perilymphatic predominant$^{\alpha}$	16 (100%)	0 (0%)	**-**
Lower zone predominant	16 (100%)	29 (94%)	0.54
More in one lung	2 (13%)	15 (48%)	**0.03**
Clustered	5 (31%)	2 (6%)	**0.04**
**Cyst morphology** N (%)			
Shape *After exclusion*$^{\$}$			
Round	16 (100%)	31 (100%)	-
Elliptical	16 (100%)	12 (39%)	**<0.001**
Irregular	16 (100%)	6 (19%)	**<0.001**
Variable	16 (100%)	8 (26%)	**<0.001**
Thin wall	16 (100%)	30 (100%)	-
Traversing vein	10 (63%)	8 (26%)	**0.03**
Multiseptated	16 (100%)	30 (100%)	-

[a]BHD – Birt-Hogg-Dubé syndrome confirmed by genetic testing; *N – number of patients; $^{\$}$total cyst number <5 excluded from analysis of location and distribution (N=57); $^{\alpha}$Perilymphatic predominant – subpleural, along the interlobular septa and perivascular cysts.

Of the 87 patients with cysts and no genetic testing, 57 (66%) had <5 cysts. The remaining 30 cases, with median [IQR] age 70 [62-74], included 19 smokers. Of those with >5 cysts, 3 refused genetic testing and 2 were deemed noneligible for testing after genetics review; all other cases were not referred for genetics review as detailed in Figure 1 of the manuscript.
